# Karnal Bunt: A Re-Emerging Old Foe of Wheat

**DOI:** 10.3389/fpls.2020.569057

**Published:** 2020-09-29

**Authors:** Santosh Kumar Bishnoi, Xinyao He, Rahul Madhavrao Phuke, Prem Lal Kashyap, Amos Alakonya, Vinod Chhokar, Ravi Prakash Singh, Pawan Kumar Singh

**Affiliations:** ^1^ICAR-Indian Institute of Wheat and Barley Research, Karnal, India; ^2^International Maize and Wheat Improvement Center, Texcoco, Mexico; ^3^ICAR-Indian Agriculture Research Institute, Regional Station, Indore, India; ^4^Department of Bio and Nanotechnology, Guru Jambheshwar University of Science and Technology, Hisar, India

**Keywords:** Karnal bunt, *Tilletia indica*, host resistance, cultivar development, epidemiology, climate change

## Abstract

Wheat (*Triticum aestivum* L.) crop health assumes unprecedented significance in being the second most important staple crop of the world. It is host to an array of fungal pathogens attacking the plant at different developmental stages and accrues various degrees of yield losses owing to these. *Tilletia indica* that causes Karnal bunt (KB) disease in wheat is one such fungal pathogen of high quarantine importance restricting the free global trade of wheat besides the loss of grain yield as well as quality. With global climate change, the disease appears to be shifting from its traditional areas of occurrence with reports of increased vulnerabilities of new areas across the continents. This KB vulnerability of new geographies is of serious concern because once established, the disease is extremely difficult to eradicate and no known instance of its complete eradication using any management strategy has been reported yet. The host resistance to KB is the most successful as well as preferred strategy for its mitigation and control. However, breeding of KB resistant wheat cultivars has proven to be not so easy, and the low success rate owes to the scarcity of resistance sources, extremely laborious and regulated field screening protocols delaying identification/validation of putative resistance sources, and complex quantitative nature of resistance with multiple genes conferring only partial resistance. Moreover, given a lack of comprehensive understanding of the KB disease epidemiology, host-pathogen interaction, and pathogen evolution. Here, in this review, we attempt to summarize the progress made and efforts underway toward a holistic understanding of the disease itself with a specific focus on the host-pathogen interaction between *T. indica* and wheat as key elements in the development of resistant germplasm. In this context, we emphasize the tools and techniques being utilized in development of KB resistant germplasm by illuminating upon the genetics concerning the host responses to the KB pathogen including a future course. As such, this article could act as a one stop information primer on this economically important and re-emerging old foe threatening to cause devastating impacts on food security and well-being of communities that rely on wheat.

## Introduction

Fungal pathogens are the leading biotic stresses of wheat (*Triticum aestivum* L.). Among these, the smut fungus *Tilletia indica* Mitra (syn. *Neovossia indica* (Mitra) Mundkur), causing Karnal bunt (KB), is an important and old disease of wheat with restricted occurrences in Asia, Africa, and North and South America (Emebiri et al., 2019a; Emebiri et al., 2019b; Gurjar et al., 2019; Singh J. et.al., 2020). The disease is seed, soil, and airborne and affects both the quality and quantity of the wheat grains. The presence of the pathogen in a region or country results in quarantine restrictions that prevents international trading of wheat grains from the affected regions (Carris et al., 2006; Figueroa et al., 2018; Pandey et al., 2019). Most wheat importing countries insist on an additional declaration from the exporting countries that the wheat consignment being traded is produced in a KB-free area (Gurjar et al., 2019). This makes KB a challenge to the grain industry as it constitutes a global non-tariff barrier to the wheat trade. Most wheat importing countries have zero-tolerance limit for the KB causal pathogen that is considered a biosecurity threat (Singh J. et al., 2020). Even if a country, due to some reason, allows wheat grain consignment from another country with a reported occurrence of the disease, such situation would inflict a significant extra cost on the importing country in the form of inspection, interception, quarantine, and disposal etc. making the importing country look for a KB-free exporter. This is evident from the fact that despite the existing regulations and restrictions, the Karnal bunt pathogen-*T. indica* is being regularly intercepted in the consignments to countries of the European Union (https://planthealthportal.defra.gov.uk/assets/factsheets/karnal.pdf). At present, KB is considered to be a disease which is staging a resurgence in the north-western plain zone (NWPZ) of India (Singh et al., 2007; Gurjar et al., 2019; Singh J. et.al., 2020) with the eastern parts of Pakistan and Afghanistan being at a high risk of an outbreak (CIMMYT, 2011). The vulnerability of specific areas in Europe and Australia has been reported in previous studies (Wright et al., 2006; Riccioni et al., 2008). This agronomically minor but quarantine major disease is a major hindrance to wheat cultivation, production, and movement from the areas of its endemism. Therefore, it is of paramount importance that wheat breeding programs in the affected and vulnerable countries augment the efforts toward the identification and development of resistant sources (Singh S. et al., 2020). In spite of the high economic importance of KB, unfortunately, resistance breeding targeting KB has never been given much priority compared to other fungal diseases of wheat like rusts and Fusarium head blight possibly owing to geographical confinement of the disease to a few countries or low direct yield loss that mostly stands between 1% and only in rare cases up to 20% to 40% (Vocke et al., 2002). Compared to rusts and mildews, the identification, characterization, and cloning of the KB resistance genes have lagged much behind (Singh S. et al., 2020). The neglect of this disease is not justified given the quarantine consequences and its widespread occurrence in different countries spread across the continents of Asia, Africa, and North and South America. These countries are not only among the highest wheat producers of the world but also house a significant proportion of food and nutritionally insecure human population of the world. Further, information on pathogen evolution and race classification (transforming the geographic pathogen isolates into genetic ones), nature and durability of KB resistance (identification and introgression of “KB-free” trait), narrow range disease surveillance, and pathogen monitoring are sporadic and cannot be considered adequate. The major stakeholders in the international wheat trade have unsuccessfully proposed deregulation of KB from the quarantine obligations citing mostly negligible yield loss attributable to the disease (Vocke et al., 2002). Taking these aspects into consideration, in this review, we highlight the need to prioritize KB resistance in wheat breeding and cultivar development programs in order to avert existing quarantine restrictions that threaten to paralyze international trade in wheat and use of it as a major staple food crop. We also analyze the challenges leading to poor identification, quantification, and introgression of KB resistance into agronomically superior cultivars. We also explore how to circumvent these KB breeding pipeline stumbling blocks through application of next-generation sequencing tools, associated gene discovery, annotation, and the use of pathogen effectors based breeding in the absence of this overtly regulated pathogen (Singh S. et al., 2020).

## History, Nomenclature and Taxonomy of KB

**Figure 1:**
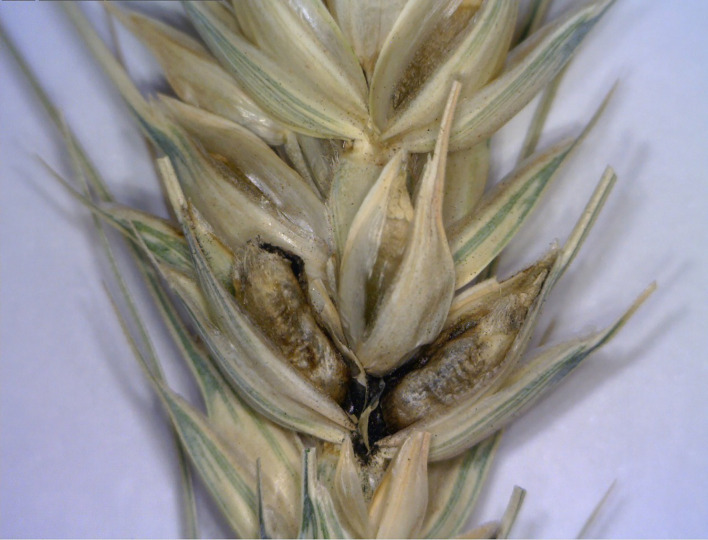
Wheat spikelets infected by *Tilletia indica*.

**Figure 2:**
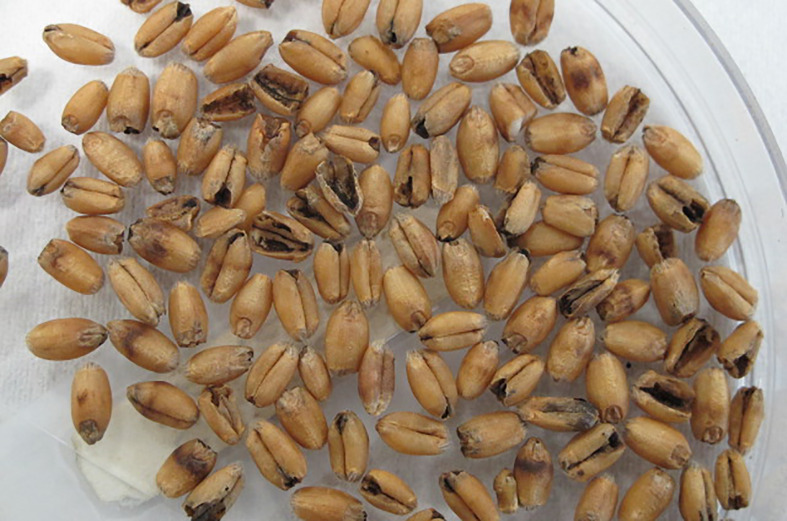
Wheat kernels infected by *Tilletia indica*.

KB was first identified by Manoranjan Mitra in the year 1931 (Mitra, 1931), in an infested experimental field at Botanical Experimental Station at Karnal, India, the name which the disease came to be known after. Interestingly, Joshi et al. (1983) reported that even before Mitra (1931), Howard and Howard had described a similar bunt of wheat from Lyallpur, Pakistan in the year 1909, but due to a lack of a type specimen that would have been used to confirm if indeed it was KB, Mitra (1931) is decisively credited with its discovery. In literature, KB is referred to by three other names: stinking bunt, new bunt, and partial bunt (Mitra, 1931; Mitra, 1937; Bedi et al., 1949) with each of these three names signifying a specific characteristic of the KB disease itself. For example, unlike other bunts, the KB infection does not cover the whole wheat ear, rather it is restricted to a few kernels within a spike (**Figure 1**) and to a part of the grain and seldom the whole grain (**Figure 2**), thereby, the name partial bunt (Pandey et al., 2019). The infected spikes emit a fetid unpleasant odor of rotten fish or dead mice caused by trimethylamine, thus, giving it the name “stinking bunt” (Mitra, 1937). Lastly, since other bunts of wheat were already known by the time KB was discovered, Mitra first named it “new bunt.” The attributes associated with the first two names are the most prominent ones in the visual field diagnosis of the KB. These three KB characteristic based names gave way for “Karnal bunt” eventually and, currently, these are rarely used.

The causal fungal pathogen of KB i.e. *Tilletia indica* (syn. *Neovossia indica*) belongs to the phylum Basidiomycota, sub phylum Ustilaginomycotina, and order Ustilaginales in the family Ustilaginaceae (Nagarajan et al., 1997), which is a family of smut fungi, containing 17 genera and 607 species in the genus *Tilletia*. The fungal pathogen causing KB has been a subject of persistent taxonomical controversies, particularly relating to its generic status as either *Tilletia* or *Neovossia* (Carris et al., 2006). Based on molecular analysis studies, Castlebury et al. (2005) concluded that the two were the same genera and the plant species assigned to them separately by Munjal (1975) had no molecular support. Since then, the genus *Tilletia* was adopted conclusively and, at present, *Neovossia indica* (Mitra) Mundkur is an accepted synonym of *Tilletia indica* Mitra.

## World Occurrence, Endemism Hypothesis, and Vulnerable Geographies

Since its discovery in the year 1931, KB remained a little-known wheat disease localized in North-West India, for four decades with almost no economic or quarantine importance realized (Duran and Cromarty, 1977; Warham, 1986). However, by the mid-seventies, the disease became of frequent occurrence in the entire northern India and, within a couple of decades, it was officially reported from other Asian countries including Afghanistan, Iraq, Nepal, Pakistan (Punjab and North-West Frontier Provinces) and Iran (Warham, 1986; Torabi et al., 1996) apart from Mexico (Sonora, Sinaloa, and Baja California Sur) (Duran, 1972), Brazil (Rio Grade do Sul) (Da Luz et al., 1993), the USA (New Mexico, Arizona, Texas, and California) (Ykema et al., 1996) and, finally, from South Africa (Northern Cape Province) (Crous et al., 2001) in the present century (https://www.cabi.org/isc/datasheet/36168). One of the reason for this rapid spread might be the sudden increase in the wheat seed movement during and post green revolution era from India to Mexico and then from Mexico to the other countries. In India, KB was an obscure disease prior to 1970s, with almost no importance given in the wheat research programs of that time. Then after the dawn of the green revolution around the mid-1960s, when the replacement of native Indian cultivars with the semi-dwarf varieties was taking place, KB incidences became unusually frequent in the North-West India. The North-West India was the cradle of the green revolution and the sudden shot up in the KB incidences could be immediately correlated with the replacement of native tall Indian wheat cultivars grown before 1960s with the Mexican semi-dwarf varieties (Joshi et al., 1983). Later studies, however, proved the crucial roles of intense irrigation and large-scale fertilizer application in the increase in intensity and number of KB incidences over entire Northern India. The green revolution mega cultivar “Sonalika” was the first one to fall prey to KB, only to be followed by the other popular varieties viz. “HD2009,” “WL711,” and “UP262” (Joshi et al., 1983; Singh and Gogoi, 2011; Saharan et al., 2016). The susceptibility of the green revolution varieties was traced to the lack of a morphological defense mechanism to KB, unlike the traditional Indian varieties, as they were formally not bred for resistance to the then economically insignificant KB disease (Warham, 1988). The importance of morpho-physical barriers such as glume pubescence in expression of field resistance to KB was recognized ever since the discovery of the disease (Fuentes-Dávila and Rajaram, 1994). In addition, Munjal (1975) and Agarwal et al. (1976) independently observed that the cultivation of Mexican semi-dwarf wheat cultivars with uniform flowering and high nitrogen fertilizer application to exploit the N-responsiveness of these cultivars were responsible for such rapid spread of the KB.

The regularity and severity of KB outbreaks between 1978 and 1979 could also be attributed to the complete replacement of the traditional Indian wheats with semi-dwarf susceptible cultivars and, consequently, the generation of high inoculum load every crop cycle. This geometric increase in KB inoculum stabilized; once the economic importance of the disease was understood and agronomic as well as host resistance measures were put in place. Although, the threat of KB becoming endemic to new geographies is still looming large. The weather simulation studies have shown that several provinces of Australia could provide a conducive climate for establishment of KB (Wright et al., 2006). The Europe has also been considered vulnerable in that according to Riccioni et al. (2008) the areas of the USA with confirmed KB presence can collectively constitute a potential “new trade pathway” for the disease to get an entry into Europe. However, the climatic dissimilarity may work against the establishment of KB in Europe (Jones, 2007).

## Economic Importance of KB: A Yield Loss vs Trade Restriction Scenario

With reports of as low as 0.01% to 1% annual yield loss, KB is categorized as a wheat fungal disease of intermediate economic significance (Warham, 1986; Wright et al., 2006; Duveiller and Mezzalama, 2009; Saharan et al., 2016) as compared to other diseases like rusts. Basically, KB is a kernel bunt and, therefore, the reduced yield is mainly because of the loss of grain weight that is by about 0.25% (Wright et al., 2006). While this yield loss might appear insignificant, the economic loss manifested through international quarantine regulations imposed on wheat grains from areas infested with KB and even the wheat crop raised in a KB endemic area, could be immense running in millions of dollars. (National Information Management & Support System, 2007; United States Department of Agriculture-Animal and Plant Health Inspection Service, 2018). Two ex-ante analyses have shown that the Australian wheat market would suffer a loss of 8% to 25% (AUD 490,900,000 per annum) if *T. indica* gets introduced in the country. The losses would mainly be attributed to the restriction from 77 wheat trading countries (Murray and Brennan, 1996; Stansbury and McKirdy, 2002; Bonde et al., 2004; Kashyap et al., 2019a). Many countries with KB endemism have suffered on economic front from these regulations and the United States Department of Agriculture (USDA) considers KB to be of minor importance given the low yield losses it causes and considers the phytosanitary and quarantine measures against KB to be unjustified. Therefore, it has advocated for the deregulation of *T. indica* as a quarantined pest. In the background of these efforts, five countries, namely Taiwan, Indonesia, Honduras, Vietnam, and Uruguay have acceded to the USDA request to deregulate the KB by lifting the quarantine regulations against it (National Information Management & Support System, 2007). Contrary to the efforts being done for deregulation of KB, a section of the European Union advocates rather stringent quarantine regime against the disease as most of the cultivars grown in European countries are considered to be susceptible to *T. indica* (Riccioni et al., 2008). Even early on, Nagarajan et al. (1997) have advocated the rationalization of the quarantine restrictions against KB which hamper the free market based on a detailed pest risk analysis with trade globalization. In our opinion, although, KB disease risk analysis reports have always considered *T. indica* a low risk pathogen, these analysis mainly focus on the yield losses that introduce a certain kind of bias in favor of KB. Therefore, it is of utmost importance to consider the economic effects of the quarantine restrictions that may arise from the introduction of the pathogen in such analysis. Further, the disease risk analysis must be based on details enough to consider climate change predictions, evolutionary potential (gene rearrangement potential which is quite high in case of *T. indica*) of the pathogen, especially its capability of virulence acquisition against available major resistance sources and, consequently, its epidemic potential in different wheat production systems in a truly globalized wheat trade scenario. Another mechanism by which KB causes significant losses is through the fungus’s capability to deteriorate the grain quality and palatability. Although, the nutrient composition of KB affected grains do not differ much from the healthy grains (Bhat et al., 1980), the infected grains have been reported to be high in ash and phosphorus content while lower in thiamine and lysine content implying that KB causes deterioration of protein quality. Gopal and Sekhon (1988) concluded that KB lowers the flour recovery, besides changing the gluten quality, particularly when the infected grains range from 1% to 5%, leading to weak dough strength. Joshi et al. (1983) did not observe any toxic effects of trimethylamine in rats, chickens or monkeys fed on KB infected wheat.

The KB infected grains are of low quality as they harbor an unacceptable smell, color, and taste and at as low as 1% infection the grains/flour becomes unpalatable (Duveiller and Mezzalama, 2009; Kashyap et al., 2018). Consequently, any KB infected wheat grain lot mostly ends up relegated to animal feeds, ultimately, fetching a significantly lower price in the market and resulting in considerable financial losses to the growers (Pandey et al., 2019; Emebiri et al., 2019a). The economic damage pertaining to the quality aspects of grains can be addressed by estimating the adverse effects of KB infection on various price affecting quality parameters of the grains. The quality aspects of wheat, particularly the gluten quality has been much worked upon lately, but information on how KB affects the gluten concentration and quality are lacking in the available literature. In addition to this data, the comparative micronutrient analysis studies of healthy and KB infected grains and the physicochemical and rheological properties of flour are also not available. More information is also needed on the possible human health effects associated with the consumption of KB infected and trimethylamine contaminated wheat kernels. Such studies could stimulate further efforts in the investment in the research on management of KB and minimizing the economic losses to the growers.

## KB Epidemiology and *T. indica* Life Cycle

The KB causal pathogen *Tilletia indica* can be soil, seed or air-borne but the incidence and intensity depend heavily on the presence of conducive environmental conditions for fungal growth (Kashyap et al., 2011; Biswas et al., 2013). A relatively cold and humid climatic regime favors KB infection mediated by production of secondary sporidia called the teliospores. The teliospores are the actual infection-causing entity that manifest the disease in the pericarp of the developing wheat grain. The literature on spore viability has been reviewed by Carris et al. (2006) and it has been reported that fungal teliospores are very hardy with high survival potential and have high viability even under very adverse conditions. In various studies, the teliospores have been reported resistant to many poisonous gases, methyl bromide, chloropicrin, hydrogen peroxide, propionic acid, ozone, and even to the low pH gastric juices of the animal’s digestive tract. Under natural conditions, the teliospores can survive in the extremities of desert and frosty climates for many years and under laboratory conditions, teliospore viability of five years under severe environmental stress conditions has been reported (Bonde et al., 2004; Carris et al., 2006). Additionally, the teliospores exhibit dormancy of one to six months before germination (Prescott, 1984), which confirms the earlier observation of Bansal et al. (1983) that germination is highest in the year-old teliospores. The dormancy of the teliospores is one potent trait which gives the *T. indica* an edge in survival. Once the period of dormancy is over and favorable conditions become available, the germination of the teliospores takes place on the surface of the soil which now are ready to infect the host wheat plant. The variability and genetic control of teliospore dormancy can potentially help in the development of a race designation system in *T. indica*, and this aspect warrants deeper investigation. A low but continuous precipitation or cloudy weather creates a high relative humidity with cold temperatures (8–20°C), and this constitutes the perfect condition for the germination and development of infection-causing teliospores (Duveiller and Mezzalama, 2009). However, it is not completely understood as to why sometimes KB incidences fail to occur in spite of the presence of favorable climatic conditions as well as the required inoculum. Therefore, the incidences and intensities of KB outbreaks are difficult to predict over the years, making the disease forecasting and management exceptionally challenging. The fine dissection of the pathogen-environmental relationship is one frontier area that should be prioritized in KB research programs for accurate disease forecasting and preparedness.

The life cycle of the *T. indica* starts when the mass of teliospores gets liberated from the infected spikes at the time of harvesting and gets spread primarily by the wind to cause the KB incidences in the next crop season. The teliospores dispersed in the soil, germinate to produce microsporidia which are also called primary sporidia, during the next crop cycle. The stubble burning has been cited to be a potent reason for long-distance teliospore travel where they have been observed at 3 km away from the site of burning (Bonde et al., 1987). This finding demands a special attention as far as wheat production system and stubble management is concerned. The teliospores have been reported to be transported by the winds over long distances and can even survive the digestive juices of the animals. However, historically, the disease has travelled across international borders or continents through infected seeds (Duveiller and Mezzalama, 2009). Therefore, the production of copious amount of very small, hardy teliospores capable of dispersal through wind, infected seeds, contaminated containers and farm machinery, human, birds, and animals across infected and non-infected areas, renders KB to be a disease of very high spread potential across geographies.

The germinated teliospores referred to as allantoid sporidia are the primary factor in epidemiology and riding on the wind or torrents of rain they arrive on the flag leaf just above the boot. Here they multiply and reach to the boot with rain-water or dew and starts the infection of spikelets (Dhaliwal et al., 1983; Kumar and Nagarajan, 1998; Carris et al., 2006). The longevity of the allantoid sporidia have been studied by many workers but the reports are contrasting to each other. The early reports by Aujla et al. (1985) and Nagarajan et al. (1997), mentioned them to be prone to desiccation and thus short-lived. On the contrary, a considerably longer viability of up to 60 days at 40% to 50% RH at 18°C temperature (Goates, 2005), and of over 46 days with temperatures above 40°C and relative humidity of 10% were reported by the later workers (Goates and Jackson, 2006; Carris et al., 2006). Subsequently, a study by Goates (2010) suggested that the sporidia have a very high potential to be dormant under the dry soil conditions with an inbuilt capability to regenerate rapidly under humid rainy conditions. If the later studies about sporidial longevity are to be considered, then it appears that germination have a little effect if any on the hardiness of these spores. However, the viability of the germinated secondary sporidia is adversely affected by reduction of the relative humidity below 76% and the rise of the temperature above 24°C (Biswas et al., 2013). *T. indica* infection starts from rachis and proceeds to glumes starting from the awn emerging stage and continuing through heading and other later stages of flowering and grain development. The seeds, which are still in the developing stage get their germinal end penetrated by the fungal hyphae (Riccioni et al., 2008). A successful infection is followed by teliospores formation in the mid layers of seed pericarp. Consequently, the endosperm contracts and these layers then split apart (Carris et al., 2006) and, eventually, replaced by the black fetid powder of teliospores. Except for some extreme susceptibility conditions, the embryo remains viable and thus germinability intact, although a significant part of the seed endosperm might be damaged (Fuentes-Dávila et al., 1996). In case of significant damage to the embryo, the seed fails to germinate, and this can be considered another mechanism by which KB can cause economic losses to the growers. It may cause low plant germination and resultantly lower yield. The teliospores are set free during harvest and are thus dispersed in air as well as on the soil surface and, in the next season, give rise to fresh infection when environmental conditions become favorable (Kumar and Nagarajan, 1998).

## Disease Symptoms and Rating

The major symptoms of KB are the presence of dark sori (black colored mass of teliospores) on the ears and the fetid smell emitted by the infected grains in field as well as in storage. However, detection of KB based on these symptoms is compromised because of their late onset, i.e. the life cycle of the pathogen is already complete when the crop is nearing harvest. Moreover, the presence of disease could be easily missed because the symptoms are not uniform and difficult to be observed in field as KB affects neither all spikes in a plant nor all the spikelets in a spike (Gill et al., 1993; Wright et al., 2006). It is the dough stage of developing wheat plant that the symptoms of KB become visible first. The infected seed parts are grey colored gradually turning black along the crease eventually destroying the scutellum and thus the grain is left with its pericarp and aleurone (Joshi et al., 1983). The symptoms and thus the infected part of the wheat kernel can be as small as a point on the germinal end to most of the kernel covered or modified into the black sori, the most typical morphologically unique characteristic of KB infections (Carris et al., 2006). And this conversion of seed into sori is in fact the visible attribute associated with the yield and quality loss of wheat produce. The precise disease scoring under screening experiment is a primary requisite for the identification of the resistant sources to KB and rating scale based on severity of symptoms devised by Aujla et al. (1989) and Bonde et al. (1996) is routinely used. The scale has four categories based on the percent bunted seed area i.e. 0= healthy (c. 5% seed bunted); 1= a point infection which is well developed (c. 25% seed bunted), 2= the crease having infection all along (c. 50% seed bunted), 3 = 3/4th of seed converted to sorus (c. 75% seed bunted), 4=sorus covers the entire seed area (c. 100% seed bunted). Riccioni et al. (2008) modified the scale by adding another category (0.1) representing inconspicuous point infection.

## Pathogen Biology and Pathogenesis

*Tilletia indica* Mitra is a hemibiotrophic and partially systemic pathogen of wheat, durum wheat and triticale. It is a heterothallic (producing opposite mating types for sexual reproduction and characterized by presence of individual self-sterility) fungus with bipolar mating (governed by a single allelic mating locus) system (Duran and Cromarty, 1977) leading to large scale genetic recombination just before infection (Gurjar et al., 2017) making the pathogenesis mechanism complex to dissect and eventually the disease difficult to handle. The sexual recombination, when compatible allantoid sporidia (+ and -) come in contact prior to the infection, is responsible for the high pathogenic as well as genetic diversity in *T. Indica* (Gurjar et al., 2019; Singh J. et al., 2020). There is a significant difference in susceptibility among wheat cultivars as far as KB infection is concerned and some are highly susceptible, resulting in a significantly greater percentage of infected kernels per spike. Some reports have also indicated relatively greater virulence of some *T. indica* isolates than others.

The race designation system unlike wheat rusts is absent in case of KB and it has been paradoxically attributed to the lack of required variability among the available isolates (Bonde et al., 1996; Datta et al., 2000). This calls for creation of a central repository of *T. indica* isolates collected from all over the globe with the aim of genetic analysis for development of a “race system” of classification. Fortunately, the genome analysis studies of *T. indica* are beginning to shed light on the molecular aspects of the pathogenicity. Gurjar et al. (2019) revealed that the genome of *T. indica* contained 97 effector linked genes, 25 virulence triggering genes, 63 loss of pathogenicity genes, and seven chemical resistance genes. Later, Pandey et al. (2019) developed proteome map of *T. indica* isolates differing in their virulence and mapped the expression of several pathogenicity factors in the highly virulent KB isolate. It was observed that the virulence proteins identified have their own functions in response to stress, host cell wall degradation and other processes crucial for a successful infection including contact, penetration, localization, establishment, signal transduction pathway activation and morphogenesis (Pandey et al., 2019). The mining of whole genome sequence and transcriptome data has been recently successfully used by Singh J. et al. (2020) for pathogenicity related genes in the *T. indica* and identified seven genes with potential roles in host penetration, infection and sporulation. This new genomic information on the pathogen will open more avenues in understanding and management of the pathogen. Nonetheless, how these genetic characteristics will relate with the changing climate is not well understood but could be critical in determining the direction of KB breeding in wheat improvement programs.

## Disease Diagnostics

In order to manage any disease, precise and early detection is critical. With *T. indica* quarantined in many countries, the effective diagnosis of KB becomes very important for free global trade of wheat (International Plant Protection Convention IPPC, 2016). The detection of KB, however, is not easy in field as well as in the stored grains. For this reason, the grains need to be removed from the spike and examined for presence of dark sori and typical fetid smell manually (Singh et al., 2016). This eye aided symptom detection may be confounded by the presence of common bunt among others which, however, affects the entire spike unlike KB (Wright et al., 2006). The laboratory diagnosis of KB is through microscopical analysis of the spores for unique morphological attributes (color and size of teliospores, cell wall structure and presence or absence of a pale sheath) which need to be confirmed through molecular techniques owing to a lesser precision of the former (Wright et al., 2006; Thirumalaisamy et al., 2011). For a successful and accurate diagnosis, the teliospores are given artificially created environmental conditions to germinate. This process is very sensitive to the conditions and viability of the teliospores is a must besides it being lengthy taking weeks together for the final report (Nguyen et al., 2019). Here, the advantage of the molecular diagnostics is that they could identify the pathogen before the formation of teliospores i.e. the inoculum for the next season and thus minimizing the risk. So far, there are four molecular diagnostic methods adopted and recommended (International Plant Protection Convention IPPC, 2016) and three of them require DNA from the germinated teliospores while one is based on multiplex real-time ITS-PCR (Frederick et al., 2000; Levy et al., 2001; Tan et al., 2009). The PCR based diagnostic methods involving the species-specific ITS primers from rDNA-ITS region have been developed, showing higher sensitivity, uniform amplification with single resolvable band compared to the mtDNA sequence-based primers (Tan et al., 2009; Thirumalaisamy et al., 2011). Although, recently reported LAMP (Loop Mediated Isothermal Amplification) assays by Gao et al. (2016) and Tan et al. (2016) also target unique sequences in the fungal mitochondrial DNA. Gurjar et al. (2017) have developed a diagnostic marker based for “in soil” detection of the teliospores. This, ideally should facilitate the agronomic management of the disease by regulating the irrigation and fertilization applications and even soil solarization of the infected fields before the wheat crop is sown. Tan et al. (2016) developed a LAMP assay based on genetic changes in *T. indica* mitochondrial genome compared to the nucleic genomes of *T. indica* and *T. walker*, which turned out to be highly sensitive, specific and cost effective. Although the genome sequence analysis of *T. indica*, *T. walkeri*, *T. controversa*, *T. caries*, and *T. laevis* identified putative genes and probes, these were validated *in silico* only. It remains unclear if they will work under laboratory conditions and on actual samples (Nguyen et al., 2019). Furthermore, Kashyap et al. (2019b) have devised the pathogenicity/virulence factors (hsp 60 and glyceraldehyde 3- phosphate dehydrogenase) based real time PCR assay for precise and rapid diagnosis of KB infection in wheat seedlings. Although under natural field conditions, the teliospores germinate and infect when the boot formation has taken place. Therefore, the relevance of this development needs to be checked under different developmental plant stages in the field.

## Host, Pathogen, and Environmental Interactions Leading to KB Development

### Host Range

*Tilletia indica* is a unique fungal pathogen reported to infect several grass species under artificial infection conditions (Warham, 1986; Gill et al., 1993; Carris et al., 2006; Gurjar et al., 2019). However, under natural conditions, the infectious capability of the pathogen is restricted only to wheat, durum wheat and triticale. Moreover, under natural infestation, bread wheat is the most susceptible host to KB while durum wheat exhibits moderate susceptibility and triticale being the least susceptible (Mitra, 1931; Warham, 1986). Interestingly, this difference in relative susceptibility of these three species vanishes under artificial infection as all three-exhibit high susceptibility to the pathogen (Warham, 1988; Wright et al., 2006). This data implies that the resistance exhibited is merely because of the presence of some morphological barriers and once these barriers are made irrelevant under artificial infection where inoculum is delivered directly inside the boot, the relativity of resistance in all the three natural host species breaks down indicating a lack of genetic difference of resistance. The host range under artificial inoculation conditions widens dramatically for *T. indica* and this includes multiple species of the genera that include *Triticum, Aegilops*, *Bromus*, *Lolium*, *Secale*, and *Oryzopsis* (Warham, 1986; Gill et al., 1993; Carris et al., 2006; Gurjar et al., 2019). The reaction of the species belonging to the genus Aegilops (the D-genome donor of *T. aestivum*) to KB is most extensively studied and *Ae. geniculate*, *Ae. sharonensis*, *Ae. peregrina*, and *T. scerrit* have been reported to harbor *T. indica* (Aujla et al., 1985; Carris et al., 2006). Apart from this, the physiological susceptibility of emmer wheat (*T. dicoccum*) to *T. indica* has also been reported (Riccioni et al., 2006) and it is one more reason to intensify the KB research globally. Although, in conclusion, it can be held that at present, the KB host of economic importance with high susceptibility under natural infestation is bread wheat and, therefore, the research and management efforts should target it primarily.

### Host Susceptibility Stages and Associated Environmental Factors

Bread wheat exhibits different degrees of susceptibility to *T. indica* depending upon the plant developmental stage and, therefore, there must be a direct relationship between these two. Despite having this understood long back, the most susceptible growth stage of wheat to a germinated teliospore is still contested. Some authors have described the heading stage to be the most susceptible (Mundkur, 1943; Bedi et al., 1949) while others proposed boot swelling to anthesis stage to be the most susceptible host growth phase. Bains (1994) compared the growth stage susceptibility of wheat plant to *T. indica* and concluded specifically that the boot emergence stage (S-2) was the most sensitive stage among all the studied ones. Kumar and Nagarajan (1998) reported stage 49 (first awns visible), to be most vulnerable to infection by secondary sporidia. The initiation of infection has largely been agreed to be from the boot stage but the last stage up to which the pathogen is capable of causing infection has been more of a range from boot to anthesis stages instead of one specific stage (Aujla et al., 1989; Kumar and Nagarajan, 1998; Pandey et al., 2019). Dhaliwal et al. (1983) found that the infection could take place as late as dough stage which was confirmed by Goates and Jackson (2006) who described that the airborne teliospores can infect wheat plant from the emergence of florets from boot stage up to soft dough developmental stage. The peak infection stage is just before anthesis when the spikes have completely emerged. The agronomic management involving foliar fungicidal sprays should coincide with this stage for an effective and comprehensive disease management. These reports have established that boot emergence or awn emergence stages are neither the most susceptible stages nor the exclusive stages for *T. indica* infection to take place. Not only this, they also asserted that the infection could take place even beyond the awn emergence stage although the airborne teliospores seem to be incapable of causing a successful infection at these later mentioned plant developmental stages. Further, morphological susceptibility tested through spray inoculation after ear emergence has been reported to be of high predictability value of the susceptibility under field conditions (Riccioni et al., 2008). This finding is of very high value in selection of a cultivar exhibiting KB resistance at physiologically most susceptible stage. The change in the weather variables from emergence of the flag leaf up to the mid-milk stage has a high correlation with the disease severity and an index named “Humid Thermal Index” has been developed based on the ratio of average afternoon relative humidity to the average daily maximum temperature pertaining to the stages mentioned (Jhorar et al., 1992).This index can be used to predict the incidences of KB based on the prevalent climatic conditions. The “Humid Thermal Index” was utilized to figure out the vulnerability of different regions to KB in Australia and Europe (Wright et al., 2006). Biswas et al. (2013) deployed various predictive regression models for predicting KB disease of wheat under Punjab (NWPZ) conditions in India and concluded that the daytime temperatures between 25°C and 30°C and night-time temperatures between 10°C and 15°C were associated with KB sporidial showering and helped in creating congenial environment for KB infection to wheat.

### Host Resistance Mechanisms to *T. indica* Infection

The genetic resistance to KB manifests through morphological barriers as well as the physiological traits. For example, the higher degree of resistance expressed by triticale and durum wheat in comparison to bread wheat is attributed to the morphological defense barriers like pubescence rather than it being physiological (Warham, 1988). The difference in degree of genetic resistance harbored by durum wheat and triticale in comparison to wheat, needs a thorough investigation and the findings can potentially bear rich dividends as far as our understanding of KB resistance mechanism in different hosts is concerned. Kumar and Nagarajan (1998) have held it that the leaf attributes, particularly the posture of the flag leaf, should also be taken into consideration as this trait might have some role to play in KB epidemiology as the flag leaf is the landing ground for the germinated teliospores. In principle, a flag leaf at an acute angle with the boot should help the allantoid sporidia to be funneled into the boot and thereby making the genotype increasingly susceptible. Gogoi et al. (2002) observed that the KB susceptible wheat cultivar “WL711” possesses some unique morphological attributes when compared to the resistant lines “HD29” and “DWL5023.” The leaf sheath, flag leaf base, glumes and rachis had significantly higher number of stomata and glumes and rachis had a low hair count relative to the resistant lines. The lower hair count implies that there is no or a very weak barrier for fungal mycelium from germinating sporidia to penetrate and establish the infection. It was also reported that the resistant lines had highly compact as well as higher number of spikelets with shorter internodes. The glume opening distance was also noted to be relatively narrow compared to the KB susceptible lines. However, Aujla et al. (1990) declared the compact arrangement of spikelets as one of the morphological features associated with KB resistance, but Singh (1992) could not observe a significant role of spike compactness in KB resistance in his artificial inoculation-based experiment. It might be because the teliospores germinate directly inside the glume through boot injection and thus surpass the morphological barrier already, which is similar to how durum wheat and triticale lose their resistance when inoculated artificially. Therefore, it can be assumed that the compactness of the spike is helpful in escaping the KB infection in durum wheat. The susceptible genotypes of bread wheat, durum wheat and triticale have a greater number of days to anthesis and thus, the early anthesis might be an escape mechanism of the host to KB (Gogoi et al., 2002). The finding that relatively more resistant durum wheat and triticale have lower glume opening (Gill et al., 1993) was confirmed by Gogoi et al. (2002) who concluded that more time for infection process to sustain was available because of more glume opening and ear emergence period in the susceptible lines. All these findings concerning the morphological barriers could be helpful in selection of genotypes with the presence of these first line of defense against KB.

## Disease Management

As with other fungal diseases of wheat, chemical and cultural management measures have been developed and recommended for KB also. However, as the disease is of sporadic occurrence and the timing of infection and damage coincide with late host growth stages, therefore, the control of KB is relatively challenging compared to other systemic smuts (Riccioni et al., 2008). Moreover, because of the low infestation, usually not up to a level to cause any significant loss, the chemical measures are generally neither applied nor comprehensively effective. Apart from this, because of the complex infection mechanism, the management of KB is difficult using cultural practices and fungicide applications (Pandey et al., 2018; Kashyap et al., 2018; Gurjar et al., 2018; Emebiri et al., 2019a). Although, application of chemical fungicides like carbendazim, triadimefon, and propiconazole as foliar spray has been found to be effective to control the KB incidences in wheat (Duveiller and Mezzalama, 2009). Nonetheless, the economic and environmental unsustainability of these fungicides overrides their effectiveness in control of KB. The efficacy of seed treatment with fungicides gets reduced significantly because the teliospores are lying down the protective covering of the pericarp of a bunted kernel. Chlorothalonil and carboxin + thiram treatment to seeds are common to control the seed borne infection of *T. indica* (National Information Management & Support System, 2007). The cultural practices including crop rotation can suppress disease development but cannot eliminate the disease because of the high survival rates of teliospores up to six years, in the soil. Mitra (1935) strongly advocated crop rotation to control the KB infestation from becoming epidemic. The rotating with non-host crops, lowering seeding rate and amount of nitrogen fertilizer, disinfecting the soil, altering irrigation and delayed planting in order to avoid humid conditions during awn emergence are recommended cultural practices to minimize KB incidences. Bedi et al. (1949) put forth two interesting findings which were the high KB incidence in irrigated fields and low KB incidences in the poorly fertilized ones. These finding demonstrated that irrigation and fertilizing the fields have a positive impact on the successful KB infestation and as such should be considered in the agronomic management of the disease. Surprisingly, experiments have indicated low incidence of KB under zero tillage as compared to conventional tillage (Saharan et al., 2016). The effectiveness of these cultural practices to minimize the disease incidence, however, is not very high. Because the pathogen is seed borne, therefore, use of disease-free seed is essential in its management. Soil mulching with polyethylene has also been proposed to be a method of reducing the teliospore viability mediated by generation of high temperature. However, in large scale commercial production of wheat and, particularly, in the developing countries, this remedy seems to be an unrealistic prospect. Kashyap et al. (2018) reported the application of plant defense activators as a promising disease management strategy. Nonetheless, till now, most effective, economical and eco-friendly recognized KB management strategy is the host plant resistance in the form of resistant wheat cultivars. In this context, the KB resistance breeding has come into the forefront of the strategies for not only disease management but also for the control of disease spread to the new areas (Fuentes-Dávila, and Rajaram, 1994; Kumar et al., 2016; Brar et al., 2018). Therefore, genetic resistance to KB is not only necessary to reduce or eliminate the associated losses but also for free global trade in wheat given the quarantine regulations imposed against it, internationally (Kaur et al., 2016).

## Steps Toward Development of KB-Resistant Germplasm

### Resistance Breeding Efforts at the CIMMYT

Breeding for KB resistance at the CIMMYT, Mexico, began in the early 1980s and since 1984, the KB screening nursery (KBSN) has been regularly distributed to different international collaborators to introgress KB resistance in their national wheat breeding programs. The CIMMYT and the National Institute for Forestry, Agriculture and Livestock Research (INIFAP), Mexico has created artificial inoculated field screening facility at the Norman E. Borlaug Experiment Station (CENEB), Obregon, Mexico, where the disease was accidently introduced and is already established. The early extensive wheat germplasm screening efforts could identify wheat lines from India, China, Brazil and the synthetic hybrid wheats (SHWs) as four major sources of KB resistance (Fuentes-Dávila et al., 1995). At present, CIMMYT is having an inventory of high yielding lines and advanced breeding lines with a good degree of KB resistance and the KB resistant material is shared on request.

### Resistant Cultivars and Challenges in Their Development

As both durum and bread wheat are susceptible to KB, therefore, initial search for genetic resistance included both. The bread wheat resistance sources, few of which have been released cultivars and other registered genetic stocks include “KBRL10,” “KBRL13,” “KBRL22,” “HD29,” “HD30,” “W485,” “W1786,” “WL3093,” “WL3203,” “WL3526,” “WL3534,” “ISD227-5,” “HP1531,” “ML1194,” of *T. aestivum* while “D482,” “D873,” “D879,” “D895” of *T. durum* which have been widely utilized for introgression of KB-free trait. The genetic resistance against KB has also been introgressed in the popular Indian wheat varieties “PBW 343” and “WH 542” through back crossing technique (Sharma et al., 2015). These varieties have been very popular historically in NWPZ of India covering a significant area. Currently, the KB resistant cultivars available globally include “Arivechi M92,” “HD29,” “HD30,” “Navojoa M2007,” “INIFAP M97,” “Munal 1” of bread wheat and “Altar C84,” “Jupare C2001,” “Aconchi C89,” “Atil C2000,” and “Banamichi C2004” of durum wheat (Duveiller and Mezzalama, 2009; Kumar et al., 2016). Although, the KB resistance breeding is difficult owing to limited variability explored for the trait, polygenic inheritance and the confounding effect of environment limiting the accuracy of field screening for identification of resistance (Dhaliwal et al., 1993; Dhaliwal and Singh, 1997; Chhuneja et al., 2008), collectively leading to limited success. The requirement of field screening of the germplasm for identification of resistance sources imposes a major constraint to the development of resistant cultivars. The field evaluation for KB has limitations pertaining to i) resource intensiveness of field screening methods in artificial creation of disease and hand inoculation of individual spikes, ii) quarantine regulations restricting the field evaluation barring few countries over the world, iii) screening against different isolates when well-defined KB isolates are unavailable leading to decreased precision and ambiguity in identification of resistance and susceptibility response iv) the field screening results are highly confounded by the effect of environment v) mechanism leading to host susceptibility is unknown and vi) quantitative inheritance of resistance complicating the segregation ratio based genetic analysis and selection (Singh et al., 2003; Brooks et al., 2006; Singh et al., 2007; Sirari et al., 2008; Kumar et al., 2016; Brar et al., 2018; Emebiri et al., 2019a). The dikaryotization of compatible mating types just before infection causes pathogen genetic recombination resulting into reduced disease incidence and high frequency of escapes even with artificial inoculation leading to confounded outcomes of the screening experiments (Dhaliwal and Singh, 1997). The selection in the early generations of a KB resistance breeding program is quite challenging because of the difficulty in establishment of a uniform disease pressure through artificial inoculation resulting into escapes and presence of incomplete resistance in populations resulting in inconsistent phenotypes (Singh et al., 1999). The screening results are highly variable, so multiple years of testing are needed to minimize the errors. Apart from the difficulties in identification of resistance sources, the incorporation of KB resistance in promising genotypes is also difficult because no single gene imparts complete KB resistance. Given the complexities and difficulties of field selection and transfer of resistance to elite cultivars through traditional breeding, the development of molecular markers closely linked to resistance QTL (**Table 1**) and eventual gene pyramiding can help in selection of resistant genotypes without screening in the field under artificial inoculation (Singh et al., 2007; Singh et al., 2012). Therefore, identifying and mapping genes conferring KB-resistance is of utmost importance for developing resistant wheat cultivars (Kaur et al., 2016). Although, many resistance sources have been identified lately, their utilization in the development of resistant cultivar has not been very successful due to a lack of genetic analysis of these sources (Brar et al., 2018). The mechanism of host-pathogen interaction remains complex making the cultivar development even more challenging. Therefore, the choice of KB resistant but high yielding wheat cultivars to the farmers is limited despite the availability of multiple KB resistance sources.

**Table 1:** QTLs reported conditioning the KB resistance trait in wheat.

S.N.	Line/genotype/Origin	Chromosome	Linked Marker/Interval/Physical position	Reference
1	Altar 84	3BS, 5AL	RFLP	Nelson et al., 1998
2	HD 29	4BL	Xgwm 538	Singh et al., 2003
3	HD 29	4BL	SNP 52bp fragment of interest (gwm538 snp)	Brooks et al., 2006
4	HD29	Qkb.ksu-5BL.1	Xgdm116–Xmc 235	Singh et al., 2007
5	HD29	Qkb.ksu-6BS.1	Xwmc105–Xgwm 88	
6	W485	Qkb.ksu-4BL.1	Xgwm 6–Xwmc 349	
7	H567.71	4B	Xgwm 6	Kumar et al., 2015
8	ALDAN	Qkb.dwr-5BL.1	Xwmc 235 and Xbarc 140	Kaur et al., 2016
9	HD29	5B	Xgdm116–Xwmc235	
10	HD29	6B	Xwmc105–Xgwm 88	
11	W485	4B	Xgwm 6–Xwmc 349	Bala et al., 2016
12	WKCBW	QKb.cim-2BL	1086228–1092041	
13	WKCBW	QKb.cim-3DL	7487658–2252592	Brar et al., 2018
14	Huirivis#1	QKb.cim-3BS1	1079551–100010977	
15	Mutus	QKb.cim-5BS2	2253589–1011847	
16	HD29	3B	IWB57185	Emebiri et al., 2019b
17	WH542	1A	IWA1644	
18	WH542	1D	IWB2650	
19	W485 1B	IW	B59865	
20	Afghanistan panel	1DL	470084827	Gupta et al., 2019
		2DL	586853396	
		4AL	656758037	
		5AS	36718388	
		6BL	500595153	
		6BS	21209894	
		7BS	45306426	
		7DL	607297738	

### KB-Resistant Sources

The earlier reports of a lack of immunity in wheat germplasm against KB were confirmed by Warham (1988). Since then the KB resistance screening has come a long way and a variety of resistance sources including nearly immune ones have been identified. The identified resistance sources for KB spans all the three gene pools including the cultivated bread wheat, durum wheat and triticale (Warham, 1986; Fuentes-Dávila and Rajaram, 1994; Dhaliwal and Singh, 1997; Sharma et al., 2005). The search for resistance sources in the post green revolution varieties started as soon as the economic importance of KB was realized mainly at CIMMYT and in India. Although, the origin of the resistant material can mostly be traced back to India, China and Brazil and the resistance in these materials has been reported to be conditioned by multiple minor genes (Chhuneja et al., 2008). The KB resistance breeding have historically involved screening of released varieties and pre-breeding involving already known resistant bread wheat lines or the wild relatives and agronomically superior lines against multiple isolates and multiple locations. In broad terms, the KB resistant germplasm can be classified in to released cultivars and genetic stocks, synthetic hexaploid wheat (SHW) and the wheat wild relatives.

#### Screening of Released Cultivars and Development of KB-Resistant Stocks

The study by Fuentes-Dávila and Rajaram (1994) early on, could confirm the susceptibility of the popular varieties “PBW120” and “PBW65” earlier reported to be KB resistant by Aujla et al. (1986) and the resistance of “WL1786,” “HD29,” and “HD30” in a different environment at Mexico, apart from identification of several new resistant lines (“Aldan/IAS58,” “Shanghai-7,” “Roek//Maya/Nac,” “Star,” “Vee#7/Bow,” and Weaver) originating from USA, Italy, Brazil, Mexico, Argentina and China at the CIMMYT. Sharma et al. (2005) confirmed the KB resistance of many of these lines apart from reporting resistance in other genetic stocks (“CMH77.308,” “H567.71/3*PAR,” “HP1531” “W485,” “CHRIS,” “Impeto,” “PREL/L1O/JAR,” “RC7201/2*BR2,” and “PF7113”) as well. These stocks are by now well established and routinely utilized for introgression in hexaploid background. Recently, Bala et al. (2015) developed a KB resistant stock named “KBRL57” from a cross involving both (“ALDAN’S”/”IAS 58” and “H567.7”) the KB resistant parents. The combining ability of these stocks is of particular importance as far as their utilization in the introgressive breeding is concerned. Compared to this, the identification of KB resistance in released cultivars such as “Eltan (Peterson, 2009),” “DBW52,” “VL829,” “VL616,” “TL2942”(I),”HS375,” “HS13,” “DDW12,” “HPW251,” “RAJ3777,” “RAJ3765,” “HPW211,” and “HPW236” (Kumar et al., 2014), Chakwal-50, Mahmood et al. (2013), Batavia, Pelsart and RAC-655 (bread wheat), Hyperno and Saintly (durum wheat) and Tuckerbox, Berkshire and Hawkeye (triticale) (Emebiri et al., 2019a) etc. is a more straightforward approach to tackle the biosecurity threat of KB.

#### Synthetic Hexaploid Wheats as KB Resistance Sources

The synthetic hexaploid wheats (SHWs) have been historically developed for introgression of biotic as well as abiotic stress resistant traits in the commercial wheat cultivars. The SHWs show resistance as well as immunity to KB due to resistance genes received from either the durum or *Ae. tauschii* (Mujeeb-Kazi, 2001; Chhuneja et al., 2008). Villareal et al. (1996) registered four SHWs lines immune to KB while Mujeeb-Kazi (2006) developed a sub set of SHW based on phenological descriptors and additional trait evaluations with most desirable combinations i.e. spring growth habit, tall, late maturity, good agronomic type and non-free threshing with a high 1000 kernel weight representing source of genetic resistance to KB. Chhuneja et al. (2008) derived homozygous introgression lines in an *Ae. tauschii* (resistant) x *T. durum* (susceptible) cross and in these lines, the KB incidence was observed to be 0% to 1.2% which was significantly lower to that in the recipient parent (10.7%) and to the highly susceptible cultivar (30%). Therefore, development of homozygous introgression lines seems to be a promising strategy for transfer of KB-free trait in the susceptible cultivars.

#### Wheat Wild Relatives as Sources of KB Resistance

Due to scanty availability of KB resistance in the primary gene pool of wheat, the wild relatives become natural candidates for resistance gene exploration. Until now, many crop wild relative species in the genus Triticum have been identified to possess KB resistance. Few of them have been utilized successfully for incorporation of genetic resistance in commercial cultivars as mentioned above. Warham (1986) reported resistance in *Ae. biuncialis*, *Ae. columnaris*, *Ae. crassa*, *Ae. juvenalis*, *Ae. ovata*, *Ae. speltoides*, and *Ae. tauschii*. In this context, *Ae. tauschii*, seems to be a very interesting case. This species is in the parental constitution of the SHWs and express both resistance and susceptibility depending on the accession in question. This implies that KB resistance is under strict genetic control in *Ae. tauschii* and highly resistant *Ae. tauschii* accessions have been identified in various studies (Sehgal, 2006; Chhuneja et al., 2008). Unlike *Ae. tauschii*, *T. urartu* was found to be completely immune while the Sitopsis section of diploid Aegilops species was reported to be devoid of resistance to KB (Dhaliwal and Singh, 1997). The genetics of the immunity of *T. urartu* and the transfer process of this to *T. aestivum* and *T. durum* need to be worked out. The importance of morphological resistance against *T. indica* was established early on and Warham (1988) proposed rye (*Secale cereale*) to be a potential source of morphological resistance to KB because of the presence of pubescence and tightly adhering glumes. Later, KB resistance was reported in *T. araraticum* (Bijral and Sharma, 1995) and *T. monococcum* (Vasu et al., 2000). KB resistance from *T. monococcum* and *T. boeoticum* was successfully introgressed in to popular spring wheat line “PBW343” and “WL711” avoiding the linkage drag of undesired genes (Singh et al., 2008). Chauhan and Singh (1994) identified barley addition lines 4H and 7H possessing a good degree of KB resistance. The homologous pairing of la,1/3, 4S, and 4L barley chromosome arms with those of the corresponding wheat homologs was proposed. Moreover, *T. aestivum* cv. Chinese spring, *T. dicoccoides*, *T. spelta* album, *T. spelta* grey, *T. tauschii*, and amphidiploids of Chinese spring with *Agropyron elongatum* (2n = 56) and *Ae. junceum* (2n = 56) have been proposed to be potential sources of KB resistance by Singh and Rajaram (2002). These proposals need to be evaluated under natural and artificial epiphytotic conditions followed by their genetic analysis to ascertain their worth as “KB-free” trait donors.

## Genetics of KB-Free Trait

### The Gene-for-gene Hypothesis for KB

Although, Bonde et al. (1996) held that as physiological specialization in *T. indica* was absent and, therefore, the gene-for-gene hypothesis should not hold good for KB resistance, however, the later studies (Datta et al., 1999; Singh et al., 1999) reported different resistance responses by different wheat genotypes to different *T. indica* isolates, indicating the presence of a possible gene-for-gene relationship. Varying number of genes operating for different KB isolates has also been reported supporting the gene for gene hypothesis for this disease. Singh et al. (1999) could differentiate the most resistant lines of durum wheat (PDW215), triticale (TL1210) and bread wheat (HD29) using different *T. indica* isolates again indicating the presence of specific gene for gene relationship among different genotypes and isolates. Singh et al. (1999) postulated that “HD29,” was having three major resistance genes against the isolate “Ni7” and two genes against “Ni8,” with one gene being common in both. Three resistance genes of “HD29,” were effective against the isolate “Ni7,” but only two were effective against “Ni8” indicating that isolate “Ni8” possesses virulence for at least one of the three resistance genes effective against “Ni7.” Although the KB isolates could not be distinguished in to distinct pathotypes and, in fact they are genetically heterogenous populations (Dhaliwal and Singh, 1997). Given this fact, the differential host response to different isolates can be explained by the difference of frequencies of virulence and avirulence alleles at different pathogenicity loci. The different number of genes providing resistance to KB against compatible monosporidial pair in different host populations is also indicative of host pathogen gene specificity and resistance against the pathogen population prevalent in a region can be screened by using a mix of pathogen isolates, particularly the ones which are exceedingly virulent (Sirari et al., 2008). This strategy involving a diverse mix of pathotypes can be helpful in breeding for durable horizontal KB resistance unless a specific extremely virulent strain of *T. indica* prompts the researchers to search for a specific gene imparting vertical resistance to a cultivar.

### Understanding the Genetic Architecture of KB Resistance

The host resistance to KB is quantitatively inherited i.e. many small effect quantitative trait loci are believed to contribute additively to the resistance as the disease is progressive and scoring is on a continuous scale (Fuentes-Dávila et al., 1995; Nelson et al., 1998; Singh et al., 2003; Singh et al., 2007). The epistatic variance is an inherent feature of the KB resistance genetics (Sharma et al., 1995) and makes the inheritance difficult to interpret in the conventional genetic analysis. Moreover, the importance of general combining ability and prevalence of additive and additive x additive gene action in KB resistance is also well documented (Morgunov et al., 1994; Tyagi et al., 2010; Kumar et al., 2016). The prevalence of additive and additive x additive gene effects means that the improvement of KB resistance in high yielding wheat cultivars should be predicted to manifest on an incremental landscape. It means that increased degree of KB resistance is expected with a unit increase in the number of favorable alleles in a cultivar. The kind of genetic interactions mentioned above to be present in inheritance of KB resistance trait also imply that the trait should ideally be highly responsive to selection. Also, the genotypes with a higher number of resistance genes should ideally be the better resistance source (Singh et al., 1995a) to utilize in a KB-free trait introgressive breeding program. Therefore, different wheat genotypes may harbor a different number of KB resistance genes and knowing their number is of very high importance to decide their inclusion/exclusion in a breeding program. Fuentes-Dávila et al. (1995) observed higher resistance in lines from the cross “Shanghai#8” and “CMH77.308” possessing three genes with dominant/partially dominant relationship than those with two or with only one gene(s). On a similar note, Singh et al. (1995a) reported that digenic genotypes such as “Luan,” “Attila,” “Vee 7/Bow,” “Star,” “Weaver,” “Milan,” “Turacio,” “Opata,” “Picus,” and “Yaco” had a higher level of resistance to KB compared with those with a single gene. These findings imply that pyramiding of multiple genes should lead to expression of KB-free trait and selection for resistance under low levels of disease in artificial epiphytotic conditions should ideally result into the accumulation of resistance genes (Singh et al., 1995a). Given this, the marker assisted selection (MAS) seems to be a technique of choice for accumulating resistance genes in a single cultivar for durable and multi-pathotype resistance not dependent upon creation of artificial epiphytotic conditions.

The resistance to KB has been confirmed to be dominant to partially dominant over susceptibility (Singh et al., 1995b; Villareal et al., 1995), and multiple genes segregating with dominant, duplicate dominant and even complementary gene action have been reported by various researchers (Morgunov et al., 1994; Fuentes-Dávila et al., 1995; Singh et al., 1999; Tyagi et al., 2010). However, as mentioned earlier, not only the number of genes conditioning the KB resistance are different in different genotypes but also their mutual interactions (dominant/recessive) are different in different genotypes. Therefore, any KB breeding program must have a comprehensive pre-breeding component aimed at understanding the genetic composition of the genotypes to be utilized as parent/donor(s) and the mutual allelic relationship of the genes present in them.

As far as number of genes conditioning KB resistance is considered, most of the genetic studies have reported one to six major genes (Morgunov et al., 1994; Fuentes-Dávila et al., 1995; Singh et al., 1995a; Singh et al., 1995b; Singh et al., 1999; Swati and Goel, 2010). However, Sharma et al. (2005) reported that the number of loci conditioning the KB resistance may be up to nine in “HD29,” “W485,” “ALDAN’S”/”IAS58,” and “H567.71/3∗PAR.” It was concluded that genetic heterogeneity of parental genotypes had no contribution to the observed deviations. Earlier also, the presence of nine loci harboring non-allelic genes in four resistant parents was reported by Fuentes-Dávila et al. (1995). KB resistance has been shown to be controlled by a single recessive gene (Bag et al., 1999), two or more genes with additive effects (Sirari et al., 2008) and two and three additive genes (Sehgal, 2006) in different genotypes. A total of three additive KB resistance genes has been reported in “HD29” (a resistant cultivar) (Singh et al., 1999; Singh et al., 2003) and in “ALDAN’S”/”IAS58” (Fuentes-Dávila et al., 1995). Sharma et al. (2005) reported that two genes for KB resistance were present in “HD 29,” “W485,” and “ALDAN’S”/”IAS 58” while three genes were present in “H567.71/3∗PAR.” Virdi et al. (2016) reported that a single recessive gene-controlled KB resistance in segregating populations of “W8627 x PBW343” and concluded that being recessive and controlled by a single gene, the resistance should not be difficult to manipulate in segregating generations.

Morgunov et al. (1994) reported that the varieties “Weaver” and “W499” were having two different dominant genes while the varieties “K342” and “Cruz Alta” had a different single allelic gene. Singh et al. (1995a) reported that in eight wheat cultivars (“Attila,” “Luan,” “Milan,” “Sasia,” “Star,” “Taracio/Chil,” “Vee7/Bow,” and “Weaver”), the resistance was digenic while it was monogenic in six lines viz. “Cettia,” “Irena,” “Turacio,” “Opata,” “Picus,” and “Yaco.” Fuentes-Dávila et al. (1995) apart from reporting of the presence of nine non-allelic genetic loci in four resistant parents also showed six resistant wheat genotypes carrying six different resistant genes. In “Pigeon,” KB resistance was conditioned by two partially recessive genes while in the other cultivars, four partially dominant genes conditioned the same. The lines “PF71131,” “Chris,” and “Amsel” were carrying only one gene while “Shanghai#8” and “CMH77.308” were carrying two genes each. Moreover, one gene was common to “PF71131,” “CMH77.308,” and “Shanghai#8,” and another to “Chris” and “CMH77.308” while different genes were carried by “Chris,” “Amsel,” and “PF71131.” Similar findings of SHWs “Chen/*T. tauschii*” with a single dominant KB resistance gene with a possibility of allelism and “Altar 84/*T*. *tauschii*” with two dominant genes and “Duerg and *T. tauschii*” with two dominant genes acting in complementary fashion were reported by Villareal et al. (1995).

It means that there should be a minimum of three genes in the four SHWs. Few observations have been made in the introgressive breeding comprising of susceptible x resistant crossing regarding number of genes and the interactions among them. In one such study, the KB-free attribute has been reported to be conditioned by two independently segregating, dominant genes in the segregating progenies obtained by crossing resistant “KBRL22” and susceptible “PBW343” (Sharma et al., 2004). Later, this finding was reinforced by Swati and Goel (2010). Therefore, the importance of the pre-breeding/genetic characterization component in KB resistance breeding cannot be over emphasized anymore.

The importance of the high heritability estimates in funneling a trait to the filial generations stands well emphasized. The reported heritability estimates for KB resistance are high and thus are indicative of a high degree of genetic determination for this trait (Gupta et al., 2019). The findings also imply that KB resistance as a quantitative genetic trait should be highly amenable to the QTL mapping. The higher estimates of heritability have also been reported by many earlier studies also i.e. heritability values of 0.75 and 0.78 were reported in two populations of wheat by Brar et al. (2018) and 0.69 on entry mean basis by Emebiri et al. (2019b). Emebiri et al. (2019b), have attributed the high heritability estimates in KB genetic studies to the precise phenotypic screening methods developed by Fuentes-Dávila et al. (1995). These protocols limit the effect of environment and thus the unexplained variations under field screening experiments and owing to this, the genetic component of inheritance could be precisely measured in high values. As per the high heritability estimates obtained and reported by various studies, it can be expected that the KB resistance is highly heritable in wheat and should be governed by a “relatively simple” genetics. Though, there is a constant need of validation of these reports through more well-structured genetic studies.

### Advances in Identification of Genomic Regions/QTL Conditioning KB Resistance

The KB resistance QTL analysis studies based on both structured as well as unstructured families and subsequent marker assisted selection (MAS) could successfully overcome the bottleneck of extremely challenging field screening and could enhance the accuracy and success of resistance identification and transfer process (Kumar et al., 2016). As far as utilization of the identified QTL is concerned, the effectiveness of conventional plant breeding approaches has been highlighted as selection for a single minor KB resistant gene is difficult because of the incomplete resistance conditioned by it and also the additive nature of the gene action (Bala et al., 2016). The identification of the QTL conditioning KB resistance in wheat has been historically attempted employing the biparental populations mostly the recombinant inbred lines (RILs), however, shortly, a shift toward QTL identification in unstructured/unrelated germplasm panel employing the Genome Wide Association Studies (GWAS) is expected, mainly in order to overcome the lengthy and complicated process of generation and maintenance of the biparental populations. The tightly linked markers can also be effectively identified employing functional genomics approaches and the ESTs.

As far as KB resistance is concerned, the QTL with substantial effects have been rarely reported. The reason might be that the parental genotypes constituting the populations were lacking a large scale variability for KB resistance or that the studies have been conducted in extremely variable environments or the seasons and thus masking the genetic effects. Although, QTL with relatively large effects are the easiest to identify and analyze, however, average effect of QTL on complex traits is a rule (Mackay, 2001). Our current understanding of the genetics of the KB comes from a few major effects QTL and thus should be considered to be insufficient warranting more QTL analysis studies.

The largest effect QTL explaining 25% of the total phenotypic variance identified and associated with the KB resistance till date is the one present on 4BS chromosome in the KB resistance stock “HD29” and associated with the SSR marker “Xgwm538” (Singh et al., 2003). It was later on converted to an SNP marker by Brooks et al. (2006) thus improving its gel-based resolution and amplification consistency. Another QTL named “Qkb.ksu-5BL.1” was found to be located on chromosome 5BL in “Xgdm116–Xwmc235” interval and explained 19% of phenotypic variance. While, the other one named Qkb.ksu-6BS.1 was mapped on 6BS chromosome in intervals “Xwmc105–Xgwm88” explaining 13% of phenotypic variance. A total of 18 genomic regions for KB resistance explaining phenotypic variation ranging from 5–20% and one consistent QTL on chromosome 2BL in a set of 339 wheat accessions from Afghanistan were identified by Gupta et al. (2019). The limitation with the major effect QTL can be that the percent phenotypic variation explained by it could be a mere overestimate because of the small sample sizes known to be “Beavis effect” (Beavis, 1994). A series of small effect QTL have been reported in these and other studies and are presented in **Table 1**. Here, it is very important to remove the bias for the detected KB QTL. It can be taken care of through comparison of the detected QTL to a distribution of expected values to know the number of missed loci (Miles and Wayne, 2008). The GWAS analysis is relatively an unexploited technique in wheat KB resistance identification and only a few studies have been published till now. Gupta et al. (2019) reported novel QTLs on chromosomes 1DL, 2DL, 4AL, 5AS, 6BL, 6BS, 7BS, and 7DL. Likewise, Emebiri et al. (2019b) detected two major clusters, one on chromosome 4B, that clustered with Qkb.ksu-4B, QKb.cimmyt-4BL, Qkb.cim-4BL, and another on chromosome 3B, that clustered with Qkb.cnl-3B, QKb.cimmyt-3BS, and Qkb.cim-3BS1.

The validation and re-validation of the GWAS analysis is required because of the high possibility of the false positives being inadvertently reported mainly due to the small panel size. These studies could be most benefitted by utilization of the gene enrichment analysis and gene ontology tools shedding light on the major role of the identified regions/genes on KB resistance, thus making their manipulation and introgression easy. We have observed that most identified genes/regions are reported to have regulatory functions (Transcription factors/transduction proteins) rather than structural one’s indicative of their potential role in the resistance mechanism.

Wheat, with a complex hexaploid genome, presents another peculiarity as far as identification and inheritance of KB resistance is concerned. Although, KB resistance has been reported in the *Ae. tauschii* –the donor of D-genome- still the D-genome seems to be the least polymorphic in different wheat lines, compared to the B-genome which is most polymorphic. This data warrants a thorough scan of the D-genome by including the *T. aestivum* lines originating from diverse geographies in the panel to have an idea about the diversity and evolution of KB resistance. Recently, Singh S. et al. (2020) reported two candidate gene hits on chromosome 4D that substantiate the hypothesis that the wheat genome and particularly the D-genome has a high potentiality of harboring KB resistance genes. It can be assumed that the non-pleiotropic KB resistance genes had not been favored by natural selection, in the global genotypes, because of the absence of the pathogen. Therefore, the wheat germplasm originating from India-the country of origin of KB-assumes great significance and should be more thoroughly investigated upon.

Another, important advance of KB research should be the improvement of the resolution of the locations of previously mapped QTL apart from making attempts to discover new ones. The utility of the small effect QTL might appear low but a comprehensive genetic model for KB resistance should include an understanding of the numbers, effect sizes, and interactions of small-effect QTL as proposed for quantitative traits by Lorenz and Cohen (2012).

## Possible Predictions That May Affect KB Disease Because of Climate Change

Just like other diseases, the occurrence of KB disease is a result of interaction between host, pathogen and environment (Chakraborty et al., 2000; Chakraborty and Newton, 2011). Therefore, changes in existing environmental conditions in areas where KB disease exists will affect its presence and severity. Some of the climatic changes predicted include increased temperature, change in precipitation, increased CO_2_ and drought (Chakraborty et al., 2000). KB is known to occur in regions of arid and semi- arid climate with hot summers, mild cold winters with some rain. It has been proposed that an increase in temperature may increase infestations by rust on wheat (Coakley et al., 1999; Chakraborty et al., 2000). This may make wheat more susceptible to other fungal diseases including KB due to compromised effector based immune responses (Selin et al., 2016). Further, the increased temperature may limit teliospores survival in the soil and, therefore, reduce inoculum density. *T. indica* is a heterothallic fungal pathogen producing haploid secondary sporidia and compatible sporidia (+and −) that hybridize after contact to become infective (Fuentes-Dávila and Duran, 1986). These recombination events may be affected by high temperature and, therefore, infection events due to a lack of infective propagules. In addition, this reduced recombination may bring in some level of stability in *T. indica* population which is always shifting, and this may stabilize evaluation and management practices. Currently, *T. indica* experiences large scale genetic recombination just before infection hence makes the pathogenesis mechanism and disease management difficult (Gurjar et al., 2017). Climate change will also come with changes in moisture and this can impact both wheat and *T. indica* in various ways. For instance, many climate changes models have predicted continuous as well as very heavy precipitation events and these can potentially be favorable for teliospore viability and germination. Initially, this may result in increased infection and disease development (Coakley et al., 1999). The increased moisture may prolong the growth period of wheat as well as annual recurrent infections mediated by the teliospores leading to higher yield losses. On the other hand, increased moisture may favor suicidal germination where teliospores may germinate in absence of host consequently reducing the inoculum density. An increase in CO_2_ levels will enhance growth rates of leaves and stem resulting into relatively dense canopies having a high humidity favoring *T. indica* infection. More rains may expand the niche of *T. indica* to other non-wheat hosts that it cannot infect naturally (Warham, 1986; Gill et al., 1993; Carris et al., 2006; Gurjar et al., 2019) and this may result in more inoculum in the ecosystem and hence higher yield losses. Higher CO_2_ may lower plant decomposition rates hence leading to increased crop residue which might result into enhanced inoculum loads at the start of crop season that may result in disease epidemics. Further elevated concentrations of CO_2_ may result in increased production infection causing fungal spores which eventually would act as inoculum in the next season. On the contrary, the higher CO_2_ levels can cause physiological changes in the host and thus can elicit host resistance mechanisms against the pathogen. The efficacy of the chemical fungicidal molecules might change with a change in the CO_2_ concentrations, relative humidity and temperature as higher precipitation will reduce efficacy of the pesticides due to reduced uptake and washdown. The elevated levels of carbon dioxide and temperatures may contribute toward accelerating the evolutionary process of *T. indica* by rendering the microclimate within enlarged canopy more favorable. It will lead to a greater number of infection cycles due to enhanced pathogen fecundity, increased pathogen population and, eventually, rapid evolution of new pathotypes. For successful colonization and infection by fungi, they secrete virulence factors known as “effectors” in order to suppress host defense mechanism and induce changes in the physiology of the plant to help the pathogen invade and establish (Selin et al., 2016). It is unclear how the different climatic changes predicted will affect the gene-for-gene model (Flor, 1956) in the context of KB as it continually evolves to make novel effectors which can dodge the plant defense better.

## Conclusion and Future Thrust

The Karnal bunt disease of wheat is of high current and potential economic importance due to its effects on quality and yield losses and the associated international quarantine restrictions levied against it. In the present era of global climate change, KB is a disease with a high potential of re-emergence in the areas where it is already endemic as well as its diffusion to new areas. This would have lasting consequences in the form of economic damages to wheat production and trade worldwide. The control of KB epidemics and not letting the disease to enter new geographies of the world constitute high priority of global wheat research and deployment of resistant cultivars is the most important step to this effect. The bottleneck in the development of KB resistant wheat varieties has have been historic, mainly because of a lack of easily combining resistance sources and their tedious identification owing chiefly to the confounding effects of the environment on the expression of quantitatively inherited KB resistance. However, these constraints can be overcome through identification, mapping of KB-resistance genes in primary, secondary and tertiary gene pools in wheat and their subsequent introgression into the elite cultivars of KB prone areas. The identification of novel sources of genetic resistance would require development of new marker system and thus novel/improvised MAS for KB resistance should be scaled up. The search for KB resistance must continue mainly on the unexplored or little explored aspects like host-pathogen interactions, pathogen race specification, gene mapping, annotation including identification of precise gene function and genomic selection in order to develop robust high yielding KB resistant wheat cultivars. There appears to be a meager possibility of de-regulating KB from international quarantine restrictions in the near future and even if it happens no country will desire to have such disease which is a permanent production and trade constraint for wheat, once established. The boom and bust cycles in KB remain by and large unreported and the effectiveness of broad spectrum resistance genes such as “Lr34” which encodes an ABC transporter and is effective against leaf rust, stripe rust and powdery mildew (Krattinger et al., 2016) need to be explored for KB as well. This strategy exploring the possibility of effectiveness of already known broad spectrum resistance genes if successful, can save important resources for a breeding program which would be required for carving a parallel trail for KB resistance breeding.

## Author Contributions

All authors contributed to the article and approved the submitted version.

## Funding

Funding received from Indian Council of Agricultural Research.

## Conflict of Interest

The authors declare that the research was conducted in the absence of any commercial or financial relationships that could be construed as a potential conflict of interest.

The reviewer GB declared a past co-authorship with several of the authors XH, RS, PS to the handling editor.
